# Differentiation of PSRA due to Group A and due to Nongroup A Streptococci in Patients with Early Arthritis and Elevated Antisteptolysin-O at Presentation

**DOI:** 10.1155/2009/286951

**Published:** 2009-03-29

**Authors:** T. L. Th. A. Jansen

**Affiliations:** Department of Rheumatology, Medical Centre Leeuwarden, P.O. Box 888, 8901 BR Leeuwarden, The Netherlands

## Abstract

A study was performed of consecutive patients presenting to a Dutch early arthritis clinic with a primary suggested diagnosis of reactive arthritis due to streptococci between April 1998 and January 2003, in a well-defined reference population consisting of 600 000 inhabitants. At 1 year after presentation out of 45 acute arthritis patients with initially an elevated antistreptolysin-O and without an alternative rheumatic diagnosis only 9 patients (20%) were not diagnosed as PSRA; 16 cases (36%) were due to NGAS, 20 cases (44%) due to GAS. The estimate of the annual incidence rate of PSRA in the Netherlands during the study was 1.26 per 100 000: 0.70 GAS-related. A diagnostic set of criteria was formulated based on the original Ayoub&Ahmed criteria by adding a serological criterium ASO/antiDNaseB ratio <1.4 and excluding a clinical criterium on chronicity/recurrency of arthritis: likelihood ratio for a positive test 7.9 [95% confidence interval (95%CI: 2.7–22.7)], for a negative test 0.06 [95%CI: 0.009–0.39].

## 1. Introduction

Streptococci in man are responsible for a variety of infections, ranging from relatively mild illnesses, such as pharyngitis and impetigo, to clinically severe pathologies such as pneumonia, septicaemia, necrotizing fasciitis or myositis. Streptococci may evoke not only bacterial/purulent but also sterile sequelae. Classically, arthritis secondary to group A streptococcal (GAS) pharyngitis is attributed to acute rheumatic fever (ARF) [1], but nowadays post-streptococcal reactive arthritis (PSRA) must be considered as well [2–5]. Dissimilarities between ARF and PSRA may exist genetically [6], on the level of humoral hyperresponsiveness [7–9], and on the level of clinical characteristics [10–16]. In individual cases diagnostic criteria may be helpful for diagnosing probably GAS-induced PSRA in particular. In 1997, Ayoub and Ahmed have proposed a set of clinical and serological criteria for the diagnosis of PSRA [17]: (A) acute-onset arthritis, symmetric or asymmetric, usually nonmigratory, (B) a protracted course or a recurrent type of arthritis, (C) poor responsiveness to salicylates/nonsteroidals, (D) evidence of antecedent streptococcal infection, (E) absence of any Jones major manifestation. Though these criteria were meant for classification and not meant for diagnostic use, they can be applied as a starter for diagnostic use for clinicians: particularly criterion (B) is unclear from the onset. Clinically, rheumatologists need to homogenize groups of PSRA patients as so far literature describes a heterogeneous spectrum of PSRA without proven prognostic implications [18]. This study reflects all new patients presenting in a five-year period with early arthritis and at presentation elevated streptococcal antibody titre suggestive of a recent streptococcal infection, and not known with a previous rheumatic condition.

## 2. Materials and Methods

### 2.1. Study Design

A cohort of patients was studied comprising consecutive patients with early and acute onset of arthritis and at presentation a serological suggestion of a recent infection with *β*-haemolytic streptococci, presenting to our outpatient clinics of rheumatology from April 1998 until January 2003: a period of 57 months. Before inclusion, other causes of arthritis were excluded: septic arthritis, rheumatoid arthritis, connective tissue disease, crystal deposition disease, reactive arthritis attributable to parvovirus B19/salmonella/shigella/campylobacter/chlamydia/gonococ-ci/spirochetes, and arthritis secondary to inflammatory bowel disease. An elevated antistreptolysin-O and/or antideoxy-ribonuclease-B titre with/without tonsillopharyngitis was a prerequisite for inclusion into the present study. All patients were according to Good Clinical Practice regularly seen on outpatient basis for followup. There was no strict study protocol for PSRA patients, but the following tests were obtained on routine basis in these patients: full blood count, erythrocyte sedimentation rate, serum creatinine, alkaline phosphatase, gamma-glutamyltranspeptidase, aspartate amino or alanine aminotranspeptidase, uric acid, IgM rheumatoid factor (IgM RF), antinuclear antibodies (ANA), C reactive protein, and urine microscopy. An electrocardiogram was obtained of all patients. An echocardiogram was obtained in cases with a cardiac murmur or with a conduction block on the electrocardiogram. Clinical evaluation was performed by a rheumatologist, that is, the number of joints involved was counted according the 36 swollen joint index (SJI).

During follow-up visits (*t* = 0, 6 weeks, 12 weeks, and every 3 month afterwards), the diagnosis was reevaluated by patient's own rheumatologist, and when after one year no alternative explanation for the arthritis had been presented, the diagnosis of PSRA became definite. This procedure was checked by an experienced clinician before entrance into this study.

### 2.2. Serological Measurements

In all patients antistreptolys-in-O (ASO) and antideoxyribonuclease-B (antiDNaseB) titres were measured simultaneously and sequentially, at least at three time points: at presentation, at 4–10 weeks after pharyngitis, and once afterwards. When a sore throat was not recollected, the titres of ASO and antiDNaseB were measured every 4 weeks during a period of 3 months. The suggestion of a recent streptococcal infection was accepted when an increased titre of ASO and/or antiDNaseB and subsequently decrease/normalization of titres were found: (1) ASO >200 U/L in adults, ASO >300 U/L in adolescents; antiDNaseB >200 U/L irrespective of age; (2) one or both titres must primarily show a significant rise and subsequent decrease: critical difference between consecutive ASO values 26% and between consecutive antiDNaseB values 14% [5]. Titres were determined by nephelometry kit from Behring (Marburg, Germany).

PSRA was diagnosed by the patient's own rheumatologist when after at least one year no alternative diagnosis could be made. Definite PSRA patients were subdivided into two separate groups, based on the ASO/antiDNaseB ratio taken at 4–10 weeks after pharyngitis/tonsillitis: ratio <1.40 a plea for GAS-induced reactive arthritis, whereas ratio >1.5, a plea for NGAS-induced PSRA [5]. Patients diagnosed by their own rheumatologist or the experienced clinician as non-PSRA are grouped together into a third separate group.

### 2.3. Analysis of Criteria

Ayoub and Ahmed have proposed criteria for the classification of PSRA which may be quite applicable for clinical use [17]: a triad regarding characteristics of arthritis, one regarding evidence of antecedent streptococcal infection, and a duplet regarding the exclusion of acute rheumatic fever: (A) acute onset of, symmetric or asymmetric, nonmigratory arthritis, (B) protracted course of arthritis or recurrent arthritis, (C) poor response of arthritis to salicylates/nonsteroidals, (D) serological evidence of antecedent streptococcal infection, (E) not fulfilling any modified Jones major criterion. The combination of this quintet of criteria is tested in our study groups. Criterion (B) is however difficult to score prospectively in a diagnostic phase from the onset in an individual patient.

A previous study from the region of Arnhem, in the central region of the Netherlands, revealed that the higher ASO/antiDNaseB ratios plea for PSRA types not due to GAS, that is, ratios >1.5 plea for nongroup A streptococcal (NGAS) infection which may be helpful particularly in cases with a negative throat culture; ratios <1.4 plea for GAS-induced reactive arthritis [5]. Therefore a serological criterion which is clear from the beginning at the diagnostic phase, is formulated for presumably GAS-induced PSRA: (F) ASO/antiDNB ratio at 4–10 weeks <1.4. A further serological criterion is formulated as (G) antiDNaseB >400 U/L which is twice above the upper limit of normal range (normal range: <200 U/L in adults).

The presented patient population is fitted into five sets of several combinations of criteria: starting with the five ((A), (B), (C), (D), plus (E)) criteria adapted from Ayoub and Ahmed, versus modification 1, that is, a sextet consisting of the aforementioned quintet plus (F), versus modification 2, that is, a sextet consisting of criteria by Ayoub and Ahmed plus (G) and modifications 3 and 4 consisting of the sextets minus (B): (A), (C), (D), (E), plus (F), and (A), (C), (D), (E), plus (G), respectively.

Using the aforementioned tests, the following test characteristics are calculated: sensitivity (Se), specificity (Sp), positive predictive value (PPV), likelihood ratio of a positive test (LR+) measured as the ratio between probability on a positive test result in GAS-induced PSRA patients versus nonindex patients ideally reaching to infinite, and likelihood ratio of a negative test (LR−) which is measured as the ratio between probability on a negative test result in GAS-induced PSRA versus nonindex patients ideally reaching to nil: using a 2 × 2 table with GAS-induced PSRA diagnosed by a rheumatologist at classification time 1 year after presentation as the golden standard, and using several combinations of criteria as index test, and in which *a* = true test positive, *b* = false test positive, *c* = false test negative, and *d* = true test negative: (legend: V[x] = square root of x; EXP[x] = 2.7182818284^x^)

1. Se = *a*/(*a* + *c*) with 95% confidence interval: Se +/− (95%CI) 1.96*V[*ac*/(*a* + *c*)^3^];
2. Sp = *d*/(*b* + *d*) with 95%CI: Sp +/−1.96*V[*bd*/(*b* + *d*)^3^];
3. PPV = *a*/(*a* + *b*) with 95%CI: PPV +/−1.96*V[*ab*/(*a* + *b*)^3^];
4. NPV = *d*/(*c* + *d*) with 95%CI: NPV +/−1.96*V[*cd*/(*c* + *d*)^3^];
5. LR+ = Se/(1 − Sp) with 95%CI: EXP[LN(LR+) +/−1.96*V[1/*a* −1/(*a* + *c*) + 1/*b* – 1/(*b* + *d*)]];
6. LR− = (1 − Se)/Sp with 95%CI: EXP[LN(LR−) +/−1.96*V[1/*c* −1/(*a* + *c*) + 1/*d* −1/(*b* + *d*)]].

### 2.4. Statistics

Intergroup comparison between probable GAS and NGAS patients was done by Mann-Whitney's two-sample test. Nonparametric Spearman's rho correlations are calculated between ESR, CRP, ASO/ADB ratio, number of arthritic joints, and number of weeks arthritis persists. *P*-values <.05 are considered statistically significant.

## 3. Results

During a period of nearly five years 45 patients with a sterile acute arthritis with at presentation a positive streptococcal serology presented to our outpatient department. All cases were sporadic, and clustering of cases was not seen. In 36 patients a significant change in streptococcal serology was observed, and in 16 a probability of PSRA due to group A streptococci could not be confirmed as they had group C or G streptococci in throat culture (*n* = 2) and a compatible high serological ratio suggestive of nongroup A streptococci (NGAS), that is, ASO/antiDNaseB >1.5. Suspicion rose in a group of 20 patients during followup that indeed group A streptococci (GAS) were the causative organisms. This indicates an estimated annual incidence rate for PSRA of 36 in our Dutch population, that is, 1.26/100 000: GAS-related PSRA (*n* = 20) indicates an annual incidence rate of 0.70/100 000, with NGAS-related PSRA (*n* = 16) 0.56/100 000.

A group consisting of 9 patients presented with arthritis plus elevated ASO but did not have a significant increase nor decrease in streptococcal antibody titres; and during followup an alternative diagnosis had to be be made. Therefore these patients are categorized into a third group of non-PSRA. During followup they were diagnosed as coincidental streptococcal carriers in rheumatoid arthritis (*n* = 3), psoriatic arthritis (*n* = 3), reactive arthritis due to Chlamydia (*n* = 1), Löfgren syndrome (*n* = 1), and Lyme arthritis (*n* = 1).

In GAS-induced PSRA, 4 out of 20 patients had positive antinuclear antibodies, without any specificity. By definition all PSRA patients have an increased ASO titre at 4–10 weeks after a sore throat/pharyngitis, since seronegative cases were excluded. In the 20 presumed GAS-induced PSRA we obtained 7 positive throat cultures (35%): all indeed consisted GAS; whereas in the 16 presumed NGAS cases 2 positive throat cultures (12%) were found: once group C and once group G streptococci. The number of arthritic joints according to the 36 swollen joint count (SJC36) is higher in the presumed GAS- than in the presumed NGAS-induced cases: means (SD) 5.2 (1.3) versus 1.9 (0.4). In GAS-related PSRA the following systemic involvement was encountered: pericarditis (1 out of 20 patients), and pneumonitis (1 out of 20 patients), whereas in NGAS-related PSRA similar extraarticular manifestations were not seen.

GAS-related PSRA patients all presented with a nonmigratory type of arthritis; 45% had arthritis which persisted for more than 3 months (by our definition protracted course), and in 10% arthritis recurred. Arthritis with characteristics protracted *or* recurrent occurred in 50%. Good response to salicilates/nonsteroidals was seen in 1 out of 20 patients. All patients had proof of recent streptococcal infection in serology, either in ASO and/or in antiDNaseB. None of the patients presented with a major Jones' criterion other than arthritis, nor fulfilled Jones' criteria on ARF. By definition all GAS-related cases presented with a low ASO/antiDNaseB ratio. Altogether 95% of GAS-induced PSRA patients fulfilled the criteria A + C + D + E + F, by including criterion B this percentage dropped to 50%. When criterion B is excluded, 75% of NGAS-induced patients meet criteria set A + C + D + E. In the 9 non-PSRA cases criteria set A + C + D + E + F is met in 33% still.

In 45 patients with acute arthritis consisting of GAS-induced PSRA in 20, NGAS-induced PSRA in 16, and other cases in 9, the following test characteristics for finding GAS-induced PSRA are calculated: sensitivity (Se), specificity (Sp), predictive value of a positive test result, that is, positive predictive value (PPV), predictive value of a negative test result, that is, negative predictive value (NPV) with 95% confidence intervals (95%CI). Also likelihood ratio of positive test (LR+) and likelihood ratio of negative test (LR−) are calculated. Prevalence of index cases, that is, GAS-induced PSRA in this population is 44%. Only 50% of patients meet A + B + C + D + E, which represents Se, 76% Sp, with predictive values: 62.5% PPV and 65.5% NPV, and likelihood ratio for a positive test 2.1 LR+. By adding additional criterion F specificity increases to 88% and LR+ increases to 4.2. By eliminating criterion B a significant improvement is obtained of sensitivity and likelihood ratios: LR+ 7.9 and LR−0.06. The additional serological criteria (F) or (G) give similar results regarding their additive diagnostic value. For further data see Table 2.

### 3.1. Correlations

Spearman's rho in the 36 PSRA patients between period arthritis persists, mean (SD) 17 (35) weeks and swollen joint count (SJC) 3.5 (4.5) joints, is 0.55 (*P* < .001). In the 20 GAS-induced cases Spearman's correlation between period of persisting arthritis and SJC is 0.66 (*P* < .005), versus 0.26 (*P* > .05) in NGAS-induced cases. Other correlations are insignificant between the period arthritis persists on the one hand and erythrocyte sedimentation rate(ESR)/C-reactive protein(CRP) on the other.

## 4. Discussion

Lancefield group A streptococci (GAS) [2–5], but also nongroup A streptococci, group C and group G [19], are supposed to be capable to evoke reactive arthritis, though proof is lacking in literature [18]. Post-streptococcal reactive arthritis (PSRA) due to GAS may well be considered part of the spectrum or possibly a forme fruste of acute rheumatic fever (ARF) which currently seems to have vanished from welfaring communities. The clinical significance of a more benign PSRA secondary to NGAS remains unclear: it is not known whether carditis and/or chorea risks in NGAS-induced PSRA are similar to the risks in patients who developed full-blown rheumatic fever. It is a challenge for clinicians to separate these entities in a prospective manner. Previously ARF and PSRA have been differentiated and correctly classified by paediaticians applying simple clinical and laboratory variables [20]. In this paper similar variables are used to define clusters of criteria to differentiate subtypes of PSRA, which enables homogeneity of studied cohorts in future. Of course some circularity in reasoning cannot be ruled out completely as most patients referred have already been diagnosed using serological criteria which are generally applied in the Netherlands due to previous publications [4, 5, 10].

A resurgence over the last decades of other post-streptococcal syndromes has been related to the spread of different possibly more virulent clones of streptococci, higher number of patients with conditions interfering with immunity, and alterations in patterns of child care [21]. In our region of the Netherlands we find a low estimated annual incidence for PSRA, only 0.70 per 100 000 inhabitants for GAS-induced PSRA. Despite the resurgence of post-streptococcal syndromes as claimed, these low incidence rates on GAS-induced PSRA stress the importance of multicentre cooperation in order to increase insight into risk profiles for developing severe sequelae such as carditis, and may increase our insight into long term prognosis.

Next to pyogenic sequelae, GAS infections are known for their molecular mimicry resulting in non-pyogenic, sterile but potentially devastating sequelae such as in acute rheumatic fever (ARF). Patients may also develop a more benign manifestation of post-streptococcal reactive arthritis (PSRA), particularly when caused by nongroup A streptococci (NGAS) a predictor for benign courses [19]. In children probably more than in adults, GAS-induced PSRA should be considered a potential predecessor of rheumatic heart disease, which is reflected in penicillin prophylaxis regimens [22]. Currently, clinicians should be capable to separate GAS-induced PSRA from NGAS-induced PSRA, and this may be relevant as presently more specific risk factors for developing a subsequent carditis are lacking. A recent analysis of PSRA cases from literature revealed a significant clinical heterogeneity of the term PSRA [18]. Prior to an evidence-based advice of therapy clarity is needed of the clinical entity of the PSRA diagnosis.

Only a weak genetic difference between patients with ARF and PSRA has been demonstrated: ARF being possibly associated with HLA DRB1*16, and PSRA with HLA DRB1*01 [6]. This could not be confirmed by Simonini et al. [23]. It is therefore doubtful whether genetics are responsible for an individual's genetic susceptibility for developing the type of post-streptococcal syndrome. Humoral hyperresponsiveness then might be hypothesized to explain the specificity of response following streptococcal infection. A high frequency of in vitro elevated D8/17 binding to B lymphocytes has been found as a susceptibility marker for developing ARF more than PSRA [7, 8, 24]. Another explanation could be in an individual's defense, for example, dependent on Toll-like receptors. Next to host factors, microbacterial factors may be important: in GAS-induced PSRA so far, the M-serotypes 9, 11, and 28 have been found, and these are M-serotypes not known from acute rheumatic fever [25].

Clear diagnostic criteria for GAS-induced PSRA may be helpful for clinicians. There is a need for criteria considering the clinical heterogeneity in the PSRA diagnosis in current literature [18]. More homogeneity may result form an additional serological criterion of high ASO titres with lower ASO/antiDNaseB ratios, to exclude nongroup A streptococci. Already in 1997 Ayoub and Ahmed proposed a set of criteria for PSRA [17]. However, some of these cannot be applied at the patient's presentation, which thwarts diagnostic applicability. In the present study, a retrospective evaluation of the potential clinical applicability of these criteria was modified from Ayoub and Ahmed based on Dutch experience. Half of the presented GAS-induced PSRA patients, however, do not fulfil the previously proposed criteria by Ayoub and Ahmed on PSRA. Particularly the criterion of a protracted course of arthritis is met in only half of presumably GAS-induced PSRA patients. It is unclear yet whether this GAS-related PSRA patient group without protracted arthritis bears a higher risk than the other PSRA subgroups to develop, for example, carditis. On the other hand, one cannot apply this feature for diagnosing purposes at presentation as clearly a certain delay in time is needed to meet this criterion. Interestingly, a protracted course was not present in any of our NGAS-induced patients, diagnosed by serological data.

Epidemiologically, in adulthood PSRA due to GAS is estimated to occur in 0.7 per 100 000 inhabitants. There may be some underdiagnosis as clearly not all suspected patients will have been referred to our department. In childhood these epidemiologic data may be quite different, probably higher. Epidemiological reasoning to prove that PSRA really exists cannot be done by using the presented data. Such proof needs to be derived from immunology. Immunologically, the classic paradigm for molecular mimicry in GAS-induced PSRA is similar as in acute rheumatic fever after infection with GAS, underscoring the existence of PSRA. However, the presence of antibodies to an antigen in the membrane of the organism, the type 5 streptococcal M protein cross-reacting with tissue, has not been convincingly demonstrated in PSRA. The assumed causal role of streptococcal infection therefore remains to be demonstrated pathogenetically.

Ayoub and Ahmed have originally proposed criteria that are applicable only in full-blown clinical situations, not specifically for diagnostic purposes from the onset; as these appear to exclude half of our presumably GAS-induced PSRA patients [17]. An additional serological criterion, that is, either the elevated antideoxyribonuclease-B or the lower antistreptolysin-O/antideoxyribonuclease-B ratio (<1.4), as described previously [5], may be applied elegantly and leads to an improved criteria set in our presented population. Also the criterion on a protracted course, resulting in excluding all NGAS-induced cases, may become abundant. In order to homogenize the PSRA patient group one might study: (A) acute onset and nonmigratory type of arthritis, (C) poor responsiveness of arthritis to salicylates/nonsteroidals, (D) evidence of antecedent streptococcal infection, (E) not fulfilling modified Jones criteria, plus (F) antistreptolysin-O/antiDNaseB ratio <1.4, or (G) antiDNase-B >400 U/L. Prospective studies in other GAS-induced PSRA patient groups are needed for validation and proving their prognostic value. For purposes of homogeneity in studies the aforementioned criteria may have some merits. For practical purposes in an individual during the diagnostic phase a serological criterion plus (A) and (C) seem to be quite applicable.

## Figures and Tables

**Table 1:** Overview of 45 patients with acute arthritis subdivided into post-streptococcal reactive arthritis (PSRA) probably due to group A streptococci (GAS) in 20, probably due to nongroup A streptococci (NGAS) in 16, and 9 non-PSRA patients. Clinical data are taken at presentation; maximum serological data are tabulated. Legend: IgM-RF = rheumatoid factor (IgM type), ANA = antinuclear antibodies.

PSRA	Presumed GAS	Presumed NGAS	Non-PSRA
Number of females/males	11/9	7/9	6/3
Mean age (SD) in year [range]	33 (11) [17–54]	37 (17) [12–55]	40 (16) [14–70]
Positivity for			
HLA-B27	1 (5%)	0 (0%)	0 (0%)
IgM-RF	1 (5%)	0 (0%)	5 (55%)
ANA	5 (25%)	0 (0%)	2 (22%)

Antistreptolysin-O (ASO)			
* * >200	20 (100%)	16 (100%)	6 (67%)
mean value (SEM)	1390 (235)	1055 (130)	1088 (360)
Antideoxyribonuclease-B (ADB)			
* * >200	20 (100%)	2 (12.5%)	6 (67%)
mean value (SEM)	2730 (450)	295 (67)	340 (90)
ASO/ADB ratio			
<1.4	20 (100%)	0 (0%)	3 (33%)
mean value (SEM)	0.62 (0.06)	6.2 (1.2)	9.4 (5.0)

Positive throat culture	7x GAS	2x NGAS	1x NGAS
Arthritic joints; mean number (SEM)	5.2 (1.3)*	1.9 (0.4)	3.4 (1.2)
monoarthritis	8 (40%)	10 (63%)	3 (33%)
oligoarthritis	7 (35%)	5 (32%)	3 (33%)
polyarthritis	5 (25%)	1 (6%)	3 (33%)
Mean duration in weeks (SD)	25 (10)*	5 (2.5)	—
exceeding 2 months	10 (50%)	5 (31%)	—
exceeding 6 months	5 (25%)	0 (0%)	—
Pericarditis	1 (5%)	0 (0%)	0 (0%)
Pneumonitis	1 (5%)	0 (0%)	0 (0%)
Erythema nodosum/multiforme	1 (5%)	0 (0%)	1 (11%)
Cholestatic hepatitis	2 (10%)	0 (0%)	0 (0%)
Tenosynovitis	4 (20%)	0 (0%)	3 (33%)

*P-value <.05 for comparison presumed GAS-induced and presumed NGAS-induced PSRA patients. GAS no. is presumed as the causative organism if the ASO/antiDNaseB ratio is <1.4; NGAS is presumed if ASO/antiDNaseB ratio >1.5.

**Table 2:** Test characteristics of three sets applied as diagnostic criteria in 45 patients consisting of 36 patients with definite post-streptococcal reactive arthritis (PSRA) due to group A streptococci (GAS: *n* = 20) and due to nongroup A streptococci (NGAS: *n* = 16), and 9 non-PSRA patients: calculations represent values of a screening test aiming to find GAS-related PSRA only. Legend: Sensitivity (Se); Specificity (Sp); Positive Predictive Value (PPV); Negative Predictive Value (NPV); Likelihood ratio (LR+) in an ideal test is optimal when ad infinitum, as it gives a ratio between probability on a positive test result in GAS-induced PSRA patients versus nonindex patients; Likelihood ratio negative test (LR−) in an ideal test is optimal when it reaches nil, as it gives a ratio between probability on a negative test result in GAS-induced PSRA versus nonindex patients. In the second line 95% confidence intervals (95%CI) are given. For criteria (A), (B), (C), and so forth see also text in Materials and Methods section.

Set	Original quintet	Sextet 1	Sextet 2	Novel quintet 1	Novel quintet 2
	Ayoub and Ahmed	Modification 1	Modification 2	Modification 3	Modification 4
	A, **B**, C, D, E	A, **B**, C, D, E, **F**	A, **B**, C, D, E, **G**	A, C, D, E, **F**	A, C, D, F, **G**
Se	50%	50%	50%	95%	95%
95%CI	28–72%	28–72%	28–72%	85–100%	85–100%
Sp	76%	88%	92%	88%	80%
95%CI	59–93%	75–100%	81–100%	75–100%	64–96%
PPV	63%	77%	83%	86%	79%
95%CI 3	9–86%	54–100%	62–100%	72–100%	63–95%
NPV	66%	69%	70%	96%	95%
95%CI	48–83%	53–85%	54–85%	87–100%	86–100%
LR+	2.1	4.2	6.3	7.9	4.8
95%CI	0.9–4.7	1.3–13.2	1.5–25.3	2.7–23.0	2.9–7.7
LR-	0.66	0.57	0.54	0.06	0.06
95%CI	0.40–1.08	0.36–0.90	0.35–0.86	0.008–0.39	0.009–0.42
